# Unmasking the Unforeseen: A Case of Radiological Tension Empyema Mimicking a Traumatic Hemothorax

**DOI:** 10.7759/cureus.48617

**Published:** 2023-11-10

**Authors:** Ali Ghaffar, Syed Ali Junaid Gillani, Syed Fuzail Imam

**Affiliations:** 1 Emergency Medicine, East Lancashire NHS Hospitals, Blackburn, GBR

**Keywords:** ct chest, chest drain, cocaine use, tension, pleural empyema

## Abstract

We present a case of a previously fit and well 28-year-old male who presented to the emergency department with respiratory distress and hypoxia four days after an alleged assault and blunt-force trauma to the chest wall. Initial clinical assessment and imaging suggested a likely diagnosis of delayed massive hemothorax associated with mediastinal shift. However, upon chest drain insertion, a large amount of pus was unexpectedly drained, leading to an immediate improvement in symptoms and restoration of mediastinal anatomy on repeat imaging. Our case illustrates that, although rare, empyemas can reach a significant volume before detection; they are capable of producing radiological and clinical intrathoracic tension configuration and can mimic hemothorax in the setting of trauma.

## Introduction

After airway management, the highest priority is addressing thoracic trauma to prevent any compromise in ventilation or hemodynamic stability. Thoracic trauma is a common presentation in the emergency department, ranging from simple rib fractures to more severe forms like massive hemothorax or open chest wall injuries [1]. In a time-pressured acute setting, a swift and accurate diagnosis can make the critical distinction between life and death for the patient. To aid in diagnosis, vital information is distilled from history, examination, radiological findings, and other bedside tests. Occasionally, findings from these investigations lack specificity, leading to overlapping results with other pathological processes, causing diagnostic uncertainty, or, worse, incorrect diagnoses.

Empyema is less frequently encountered in the emergency setting. Typically, it follows an indolent course, often developing after a recent bacterial chest infection. The clinical presentation typically includes progressive worsening of pre-existing chest infection symptoms, weight loss, night sweats, and signs of sepsis [2].

Literature research reveals limited reporting of asymptomatic development of large empyemas or empyema mimicking the hallmark features of massive hemothorax in the trauma setting, making our case unique.

## Case presentation

A 28-year-old male presented to the emergency department with chest pain, hypoxia, and progressive shortness of breath after an alleged assault-related chest wall injury four days prior to the presentation. The attack primarily involved repeated blows to the left side of the chest wall. The patient was tachypneic and tachycardic and required high-flow oxygen. His blood pressure and temperature were 122/63 mmHg and 37.2°C, respectively.

**Figure 1:**
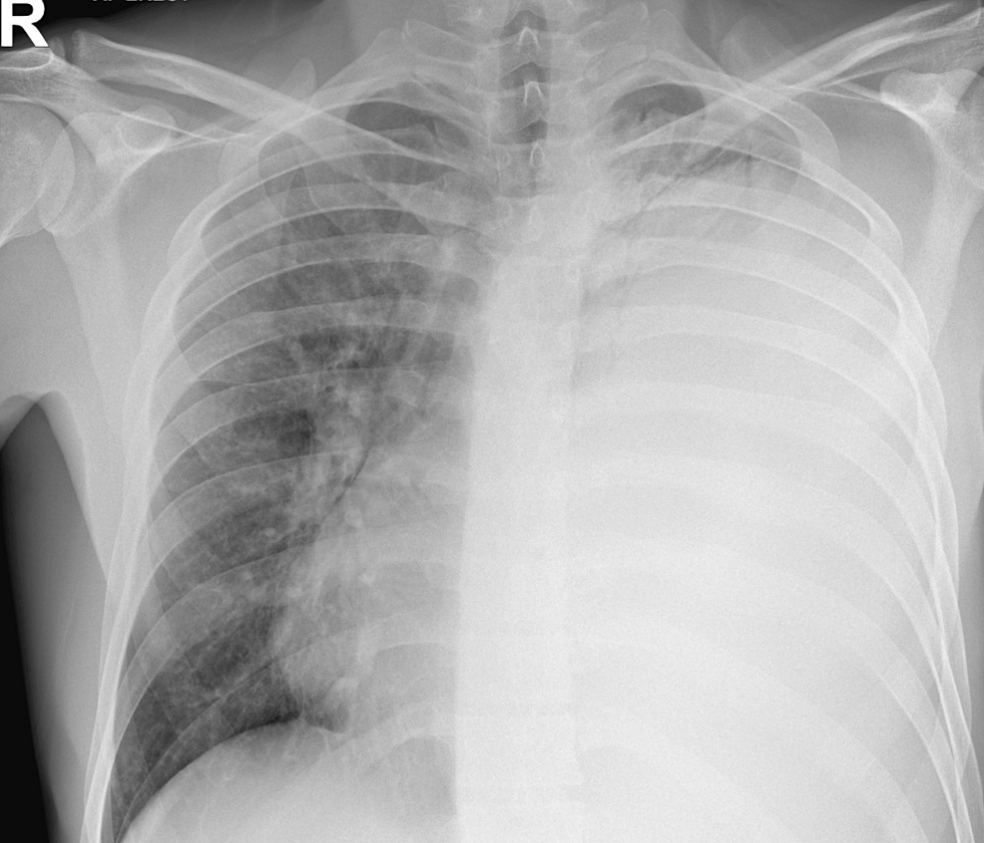
Initial chest X-ray at presentation shows homogenous opacification of almost the entire left hemithorax with rightwards mediastinal shift.

Clinical examination revealed left chest wall tenderness, rightward tracheal deviation, absent air entry on the left side of the chest, and dull percussion notes on the left hemithorax. A chest X-ray showed ipsilateral airspace opacification of almost the entire left hemithorax with displacement of the mediastinal structures to the right (Figure 1).

An initial diagnosis of delayed massive hemothorax was made. While preparing the chest drain equipment, an urgent CT scan of the thorax was acquired to locate the source of bleeding. As the blood loss was estimated to be more than 1500 mL as suggested by the mediastinal shift on imaging, blood products were requested, and preparations were made for a potential thoracotomy as per the Advanced Trauma Life Support (ATLS) guidelines [1].

**Figure 2:**
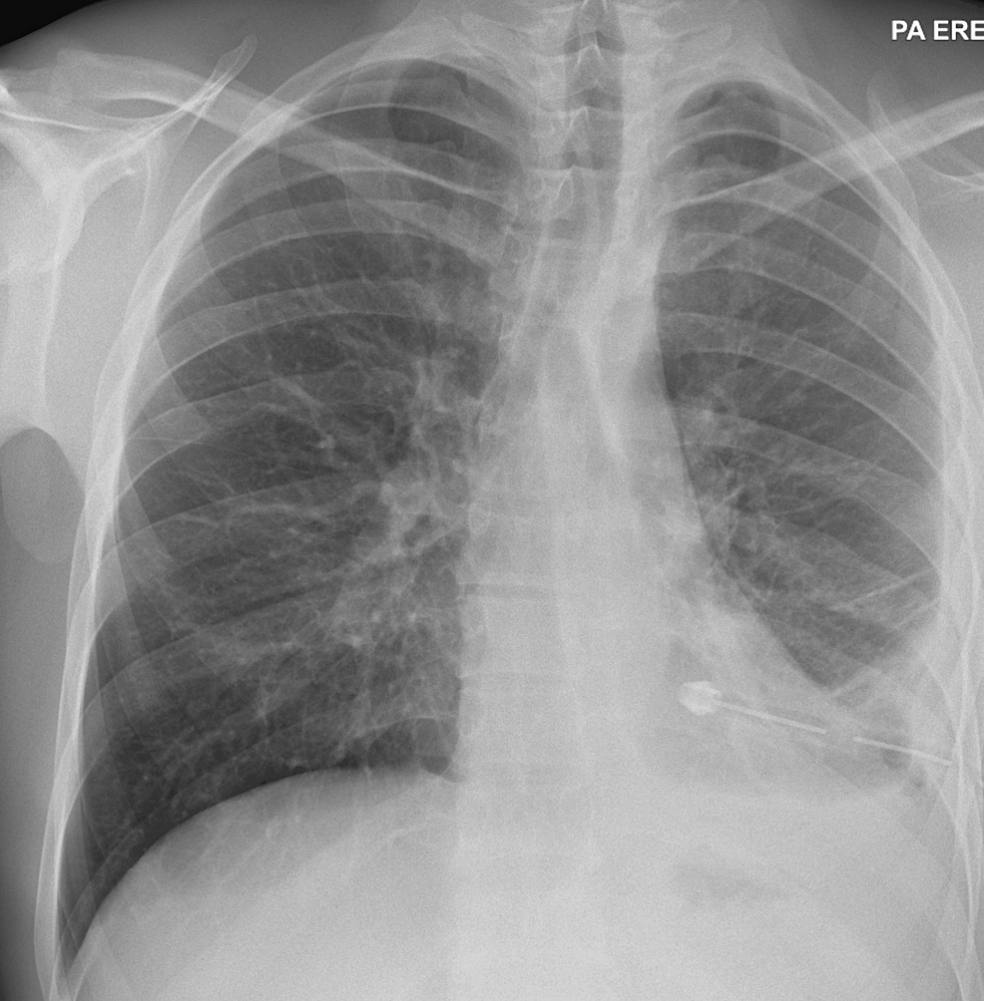
Chest X-ray after chest drain insertion shows interval reduction in the left hemithorax opacification and restoration of the mediastinal alignment.

A large-bore surgical chest drain was inserted awaiting the CT report. To the team's surprise, the drain produced a large volume (3900 mL) of frank purulent pus under high pressure, with no blood mixed in. Following chest drain insertion, there was an immediate recovery in symptoms and restoration of the mediastinal anatomy on repeat imaging (Figure 2). Intravenous broad-spectrum antibiotics were subsequently initiated.

**Figure 3:**
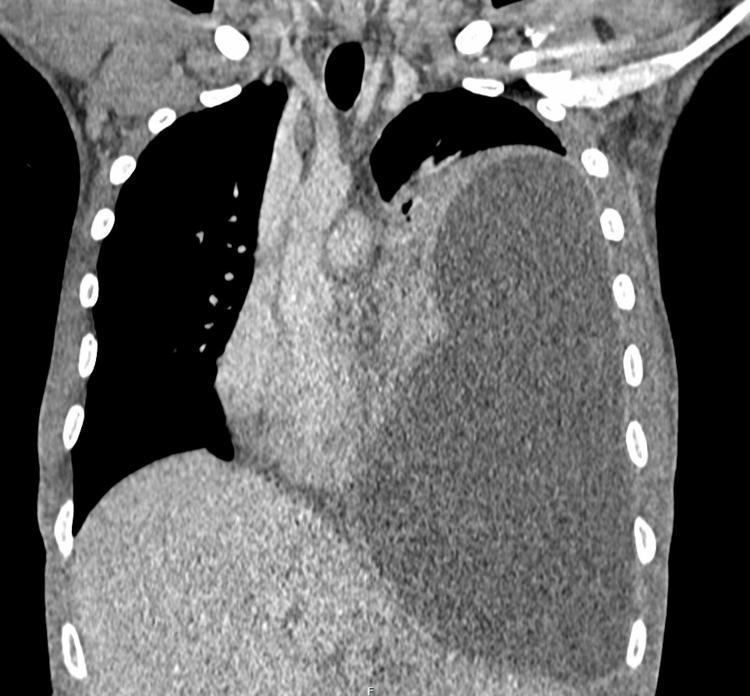
Coronal view CT image of the thoracic cavity shows a fluid collection in the left hemithorax.

**Figure 4:**
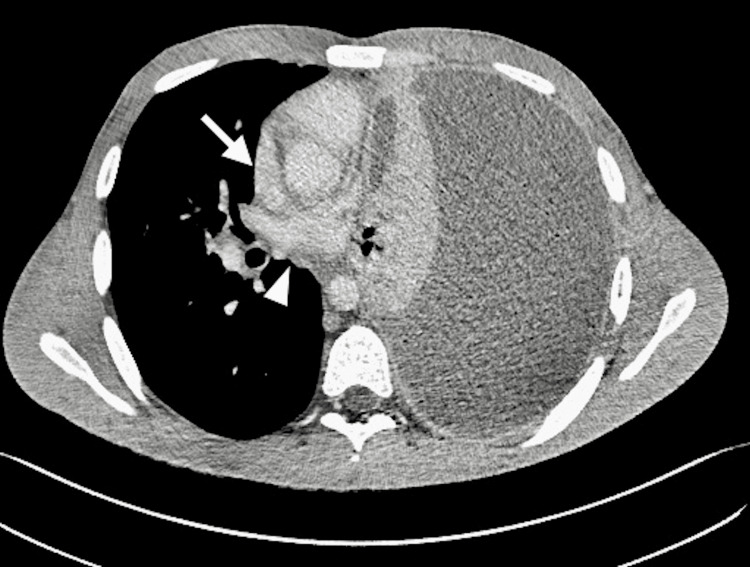
Axial view CT image of the thoracic cavity shows partial effacement of the right atrium (arrow) and left atrium (arrowhead).

**Figure 5:**
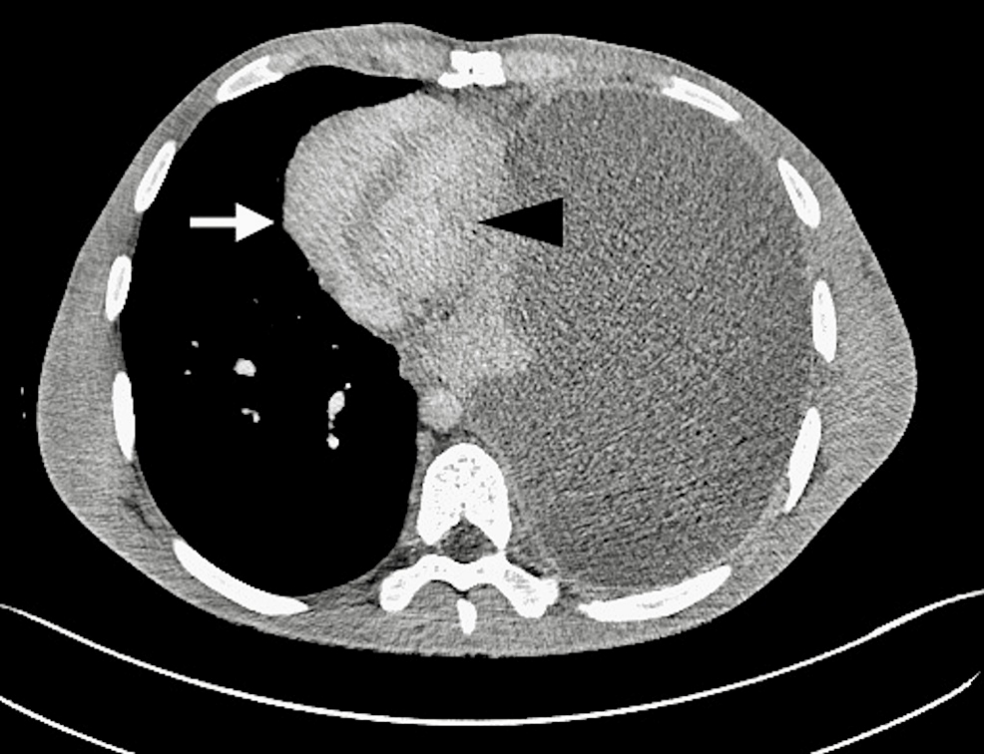
Axial view CT image of the thoracic cavity shows partial effacement of the right ventricle (arrow) with relatively preserved left ventricle morphology (arrowhead).

The CT scan report confirmed the clinical findings as a large volume collection of proteinaceous mean attenuation fluid along with edge enhancement, consistent with a large empyema. Of note, there was ipsilateral lung collapse, contralateral mediastinal displacement, and ipsilateral diaphragm inversion due to increased left hemithorax pressure (Figure 3). Increased intrathoracic pressure resulted in apparent distortion of heart morphology, with partial effacement of the right atrium, right ventricle, and left atrium. However, the left ventricle luminal diameter remained relatively intact (Figures 4, 5). No occlusion of large vessels, rib fractures, or active vascular bleeding was observed.

The white cell count was reported as 18.2 x 10^9/L and C-reactive protein was measured at 130 mg/L. Pleural fluid lactate dehydrogenase was 31328 IU/L and pH was 6.8. Pleural fluid microscopy showed no evidence of malignancy and grew *Streptococcus pneumoniae*.

The patient denied recent chest infections or invasive procedures but admitted to having snorted cocaine once a few weeks ago. There was no significant past medical history or any family history of immunodeficiency disorders. Tests for hepatitis, HIV, tuberculosis, and COVID-19 were all negative. An echocardiogram was carried out to assess for any valvular vegetation, which was unremarkable.

The patient made a satisfactory recovery after two weeks of inpatient stay for intravenous antibiotics.

## Discussion

While the term "tension" has traditionally been associated with tension pneumothorax, defined as a life-threatening condition of expanding air volume within the pleural space leading to hemodynamic compromise, there have been rare instances where large-volume empyemas have produced clinical signs resembling intrathoracic tension, justifying the use of the nomenclature "tension empyema" [3-5].

Given the high mortality rate and the need for urgent treatment, the diagnosis of intrathoracic tension typically relies on promptly recognizing clinical signs rather than seeking radiological confirmation. The "classic" clinical signs include hypoxia, absent unilateral breath sounds, respiratory distress, and hemodynamic compromise. However, since these clinical findings have often been described based on animal models, they may not always consistently mirror human physiology. Due to a lack of uniformity in the available literature regarding which features must be present and to what extent to qualify for the diagnosis of intrathoracic tension in humans, this syndrome lacks a reference standard [1,3,6,7].

Radiological manifestations, such as ipsilateral lung collapse, inversion of the ipsilateral diaphragm, and contralateral mediastinal deviation, have also been used to describe intrathoracic tension. In our patient's case, the CT report confirmed the presence of these anatomical changes [8]. Furthermore, the cross-sectional images showed effacement and partial collapse of the right heart and left atrium due to extrinsic compression of the mediastinum, likely affecting venous return and cardiac output (Figures 3-5). Studies have shown compensatory mechanisms, primarily tachycardia and tachypnea, may delay the development of hypotension in intrathoracic tension until the pre-terminal stages, with some adults exhibiting only respiratory signs without hemodynamic compromise [3]. Our patient displayed both tachycardia and tachypnea. The maintenance of normal blood pressure alongside radiological tension configuration can likely be attributed to robust compensatory mechanisms in this young, healthy adult, allowing adaptation to physiological stress.

One proposed definition of intrathoracic tension is a significant respiratory or hemodynamic compromise that reverses on thoracic decompression [3,6]. This was also observed in our patient's case upon chest drain insertion.

Confirmation bias, defined as an inclination to give greater weight to information that supports an initial hypothesis at the expense of contradictory evidence, likely played a role in the initial diagnosis of traumatic hemothorax [9]. Clinical and X-ray findings corresponded with the reported timeline and site of trauma. A large volume of empyema is likely to have developed over weeks to months rather than days. However, our patient only became symptomatic four days after the chest wall trauma. This, coupled with the absence of any recent chest infection and apyrexia at the time of presentation, led the clinicians to reach the diagnosis of a likely delayed traumatic hemothorax. Delayed development of trauma-related hemothorax is rare but has been reported in the literature [10].

The development of symptoms only after trauma can likely be explained by shallow breathing due to post-traumatic painful splinting, which now complicated the previously subclinical large-volume empyema. The drained pus was clear of any blood, making traumatic hemorrhage an unlikely contributing factor in the development of intrathoracic tension.

Regardless of the cause, once the intrathoracic tension morphology was diagnosed, a large-bore chest drain was inserted to decompress the intrathoracic pressure. This step represents a common intersecting treatment point for both hemothorax and empyema and underscores the importance of following the ATLS A-E assess-and-treat algorithm in acutely unwell trauma patients [1]. Furthermore, this case emphasizes the importance of being proficient with the potentially life-saving skill of chest drain insertion as a frontline clinician.

Pleural fluid grew *Streptococcus pneumoniae*, which is a frequently observed pathogen in cases of community-acquired empyema. Typically, the development of empyema is attributed to parapneumonic or iatrogenic origins [2]. In our patient's case, extensive investigations to identify the infection source returned negative results. There was no history of previous similar chest wall trauma to suggest an infective transformation of an existing hemothorax. The patient disclosed a single instance of snorting cocaine a few weeks before the presentation. Although literature documents the adverse effects of snorting cocaine on nasal and adjacent structures, to our knowledge, there are no reports of the development of pleural empyema in the absence of pulmonary infection and with one-off cocaine use, making our case unique [11].

## Conclusions

To conclude, our case highlights that empyemas can reach considerable sizes before manifesting clinically, may arise without a typical etiology, and have the potential to generate a tension-like morphology. Clinicians should maintain awareness of and strive to prevent confirmation bias, especially when influenced by an anchor, for example, trauma, as in our case. In situations with atypical presentations featuring overlapping signs of various pathologies, particularly under time pressure, it is crucial to keep a broad range of differential diagnoses in mind.
